# Oliceridine used for patient-controlled analgesia on postoperative quality of recovery in patients undergoing laparoscopic gynecological tumour resection: a randomized clinical trial

**DOI:** 10.1080/07853890.2026.2714613

**Published:** 2026-08-12

**Authors:** Chunyan Li, Xinna Luo, Yanli Du, Jinrui Wei, Yan Qiu, Wanyun Ge, Fei Lin

**Affiliations:** a Department of Anesthesiology, Guangxi Medical University Cancer Hospital, Nanning, Guangxi, People’s Republic of China; b Department of Anesthesiology, Liuzhou Worker’s Hospital, Liuzhou, Guangxi, People’s Republic of China

**Keywords:** Oliceridine, μ-opioid receptor biased agonists, patient-controlled analgesia, postoperative quality of recovery, enhanced recovery after surgery

## Abstract

**Background:**

Oliceridine, a novel biased μ-opioid receptor agonist, is widely used perioperatively, yet limited data exists regarding its impact on postoperative quality of recovery. This study investigated the effect of oliceridine-based patient-controlled intravenous analgesia (PCIA) on postoperative quality of recovery among patients undergoing laparoscopic gynecological tumour resection.

**Methods:**

Ninety‑four female patients scheduled for elective laparoscopic gynecological tumour resection were included. Patients were randomized to two groups: oliceridine group (loading dose 1.5 mg, PCIA 0.55 mg/kg) or sufentanil group (loading dose 10 μg, PCIA 3 μg/kg). The primary outcome was the Quality of Recovery-40 (QoR-40) score on postoperative day 1. The secondary outcomes included the QoR-40 score, the numeric rating scale (NRS) pain score, the Hospital Anxiety and Depression Scale-Anxiety (HADS-A) score, the Fatigue, Resistance, Ambulation, Illness and Loss of weight (FRAIL) index and adverse events within 3 postoperative days.

**Results:**

Higher QoR-40 scores were found in the oliceridine group on postoperative day 1 (182.9 ± 3.1 versus 177.5 ± 3.9, *p* < 0.001). Compared with the sufentanil group, the oliceridine group showed better QoR-40 scores within 3 days after operation. No significant differences were observed in NRS pain scores or HADS-A scores between the two groups (all *p* > 0.05). However, the median FRAIL score in the oliceridine group was lower on postoperative day 2 (*p* = 0.018).

**Conclusion:**

Oliceridine used in PCIA improves early postoperative recovery quality of patients undergoing laparoscopic gynecological tumour resection. It provides analgesic effect comparable to sufentanil and lowers incidences of postoperative frailty, nausea and vomiting.

**Trial Registration:**

Chinese Clinical Trial Registry, ChiCTR.org.cn, identifier: ChiCTR2400094271.

## HIGHLIGHTS

- The impact of oliceridine on postoperative recovery quality remains unclear in patients with gynecological tumours.
- Oliceridine implemented in patient-controlled intravenous analgesia improves postoperative recovery in gynecological tumour patients.
- Oliceridine implemented in patient-controlled intravenous analgesia reduces postoperative adverse events in patients with gynecological tumours.

## Introduction

Cancer continues to pose a significant threat to global public health, particularly for women [1]. The global annual incidence of gynecological cancers approximates 1.4 million, with 680,000 associated mortalities [2]. Cervical, endometrial and ovarian cancers are the three most prevalent malignancies of the female reproductive system, with their incidences rising year by year and onset ages trending younger [3]. Surgical resection is the main treatment for gynecological cancers [4]. Adjuvant chemoradiotherapy can improve patients’ 5-year overall survival rates, yet it frequently induces severe gastrointestinal adverse reactions including intractable nausea and vomiting [5]. Laparoscopic surgery has largely replaced open laparotomy for gynecological malignancies [6]; nevertheless, postoperative pain still constitutes a prominent clinical challenge [7]. Poor perioperative acute pain control can significantly raise the risks of postoperative complications, extended hospital stays and mortality [6]. Uncontrolled pain and its related side effects further hinder patients’ postoperative recovery.

Opioids such as morphine, hydromorphone and fentanyl are the core drugs of choice for postoperative pain management [8], and sufentanil is the preferred and most commonly used drug for postoperative patient-controlled intravenous analgesia (PCIA) in hospitals across China [9]. Opioids exert potent analgesic effects by binding to μ, δ and κ opioid receptors distributed in the peripheral tissues and central nervous system, thereby inhibiting nociceptive signal transmission [10]. However, opioids can cause multiple adverse reactions, including abdominal distension, nausea and vomiting, respiratory depression, pruritus and constipation [11]. Alongside their marked addictive potential, the clinical use of these opioids is greatly limited and brings considerable public health risks [12,13].

Oliceridine, a novel biased μ-opioid receptor agonist, has been approved for marketing by the U.S. Food and Drug Administration. Its unique mechanism, characterized by selective G protein-coupled receptor activation and attenuated β-arrestin recruitment, may reduce opioid-related adverse events (ORAEs) while maintaining analgesic potency comparable to conventional opioids, thereby expanding its clinical applicability [14,15]. As a Schedule II controlled opioid, oliceridine has inherent abuse potential as supported by preclinical and clinical evidence [16]. At equianalgesic doses, its rewarding effects are similar to those of morphine, and its biased agonism mechanism cannot fully eliminate addiction risks [17]. Nevertheless, oliceridine demonstrates a favourable tolerability profile [18], and the risk of addiction remains manageable when administered intravenously for short-term perioperative use in hospitals [19]. Multiple studies have confirmed that postoperative oliceridine administration reduces the incidence of vomiting and decreases the requirement for rescue antiemetics [20–22]. Compared with morphine, oliceridine does not elevate the risk of respiratory depression in elderly and obese patients, significantly lowers the incidence of ORAEs and delivers superior analgesic efficacy [23,24].

Most existing clinical trials of oliceridine have centred on orthopaedic, thoracic and gastrointestinal procedures, yet evidence for patients receiving laparoscopic gynecological tumour resection is still limited [25–27]. Females are susceptible to ORAEs [28]. Patients with gynecological malignancies exhibit reduced physical tolerance following radiotherapy and chemotherapy, with further diminished responsiveness to analgesic therapy [29]. Laparoscopic resection for gynecological malignancies is frequently associated with persistent moderate-to-severe postoperative pain [7]. Conventional opioid analgesics tend to induce ORAEs including nausea, vomiting and respiratory depression, which hinder postoperative recovery and reduce treatment adherence [30].

This prospective randomized controlled trial aimed to investigate the effect of oliceridine-based PCIA on the quality of postoperative recovery in patients undergoing laparoscopic gynecological tumour resection. Postoperative pain scores, anxiety scores, frailty scores and the incidence of adverse events were designated as secondary outcomes. These indicators allow comprehensive assessment of the overall clinical benefits of this analgesic regimen and enable the proposal of safer, more effective perioperative pain management strategies for this patient cohort.

## Materials and methods

### Study design and setting

This prospective, single-centre, randomized, double-blind controlled clinical trial was conducted from January to June 2025 in the Department of Anesthesiology and Gynecology Wards of Guangxi Medical University Cancer Hospital. The first participant was enrolled on January 5, 2025, and statistical analyses were finished in August 2025.

### Ethical approval

The protocol was approved by the Ethics Committee of the Affiliated Cancer Hospital of Guangxi Medical University (No. KY2024554, 16 October 2024) and registered with the Chinese Clinical Trial Registry (ChiCTR2400094271, 19 December 2024). Written informed consent was obtained before enrolment. This study complied with the Declaration of Helsinki and the 2010 CONSORT statement.

### Study population

Eligible participants were patients aged 18–65 years with American Society of Anesthesiologists physical status II or III, scheduled for elective laparoscopic gynecological tumour resection, and with a body mass index of 18–30 kg/m^2^. Exclusion criteria included: (1) severe cardiopulmonary dysfunction; (2) abnormal electrocardiogram, such as prolonged QT interval; (3) neuropsychiatric disorders or a history of neurological or psychiatric diseases; (4) chronic pain, neuropathic pain, or other conditions requiring prolonged use of analgesics, or a history of alcohol abuse; (5) severe infections or advanced gynecological tumours; and (6) visual or hearing impairments.

### Randomization and blinding

Using a random number table, patients underwent 1:1 randomization to the oliceridine group or the sufentanil group. Allocation sequences were placed in sequentially numbered opaque sealed envelopes and remained concealed until patients were transferred to the postanesthesia care unit (PACU). Outcome assessors, data analysts and patients were blinded to the group assignment.

### Anaesthesia and postoperative analgesia management

On the day before the operation, eligible candidates were screened and baseline data were collected by an independent investigator. Standard monitoring protocols were established immediately upon patient arrival in the operating room, including ECG, invasive arterial blood pressure, pulse oximetry and bispectral index (BIS, Covidien) monitoring.

General anaesthesia induction was performed using 0.15 mg/kg remimazolam, 0.5 μg/kg sufentanil, 0.2 mg/kg cipepofol and 1 mg/kg rocuronium in all enrolled patients. Tracheal intubation was performed once BIS values dropped below 60, followed by controlled mechanical ventilation via an anaesthesia machine. Anaesthesia was maintained via continuous intravenous infusion of cipepofol (0.3–1 mg · kg^−1^ · h^−1^) and remifentanil (0.1–0.3 μg · kg^−1^ · min^−1^), supplemented with 1% sevoflurane inhalation, to keep BIS values between 40 and 60. Neuromuscular blockade was maintained by intermittent injections of rocuronium as needed. Vasoactive drugs were given when necessary to maintain hemodynamic stability throughout anaesthesia.

After the operation, all patients were transferred to the PACU and received 12.5 mg dolasetron for antiemetic prophylaxis; PCIA was activated after tracheal extubation. The oliceridine group received a 1.5 mg loading dose, followed by a PCIA of 0.55 mg/kg oliceridine and 12.5 mg dolasetron diluted in 150 mL normal saline (initial bolus 2 mL, background infusion 1 mL/h, demand bolus 2 mL, lockout 15 min, maximum hourly rate 15 mL). Per the product label, the maximum daily oliceridine dose was capped at 27 mg. The sufentanil group received a 10 μg loading dose, with a PCIA of 3 μg/kg sufentanil and 12.5 mg dolasetron (same administration parameters as the oliceridine group). Both PCIA regimens were administered within 3 postoperative days. Patients were instructed to activate the PCIA button if their Numerical Rating Scale (NRS) score was ≥4, either in the PACU or on the ward. Rescue analgesia was administered if the NRS remained ≥4 after effective compression of PCIA or exceeded 7 at any time.

### Data collection

All scale assessments were performed by outcome assessors Yanli Du and Chunyan Li. All involved clinical staff completed standardized training prior to trial initiation. On the day before operation (T0), baseline assessments were completed in the ward examination room. On the day of operation (T1), the postoperative assessment was performed in the PACU two hours after extubation through patient interview. Follow-up assessments were conducted at 10:00 am on postoperative days 1 (T2), postoperative days 2 (T3) and postoperative days 3 (T4) on the ward; all questionnaires were filled out by patient self-reporting.

### Measurement

The primary outcome was quality of recovery, as assessed by the 40-item Quality of Recovery (QoR-40) score on postoperative day 1 (T2). The QoR-40, a validated instrument assessing recovery from anaesthesia and operation, assesses 40 items across 5 distinct dimensions of recovery: physical comfort, physical independence, emotional state, psychological support and pain. The score for each item is between 1 and 5, with an aggregate score of 200 (best quality) [31]. Secondary outcomes were assessed at T0–T4, including the QoR-40 scores; the 10-point numeric rating scale (NRS) for pain (from 0 [no pain] to 10 [worst pain]) [32]; the anxiety subscale of the hospital anxiety and depression scale (HADS-A) for anxiety scores (from 0 [no anxiety] to 21 [severe anxiety]) [33]; and the Fatigue, Resistance, Ambulation, Illness and Loss of weight (FRAIL) index for frailty assessment (from 0 [robust] to 5 [most frail]) [34].

All perioperative adverse events (AEs) were recorded from T0 to T4, including respiratory AEs such as hypoxaemia; non-respiratory AEs, including hypotension, fever and dizziness; and gastrointestinal AEs, including nausea, vomiting, abdominal distension and constipation. If moderate or severe adverse reactions related to the study drug occurred, investigators (Chunyan Li) would deliver timely interventions and treatment, document relevant data, and submit reports to the Data Monitoring Committee.

### Statistical analysis

Previous multicenter trials have established that a 6.3-point change in the QoR-40 score after perioperative interventions corresponds to the minimal clinically important difference [35]. The standard deviation (SD) was 11.4 according to the preliminary experiment in this patient population. With α = 0.05 (two-sided) and a power of 0.8, the required sample size per group was 42 by using PASS, version 15.0 (NCSS). Accounting for a 10% loss to follow-up, we aimed to enrol 47 patients in each group.

All analyses were performed following the intention-to-treat principle with SPSS 25.0 (IBM Corporation). Patient characteristics were compared between the groups. For this comparison, the Shapiro–Wilk test was applied to assess normality. The independent samples *t* test was used for normally distributed data, and the Mann–Whitney *U* test was performed otherwise for nonnormally distributed data. Continuous variables with normal distribution were presented as means and standard deviation (SD), and non-normal continuous variables were represented by median and interquartile range (IQR). Categorical variables were expressed as frequencies and proportions and analyzed using the χ^2^ test or Fisher’s exact test. Relative risk (RR) and 95% CIs were calculated to quantify differences in dichotomous outcomes.

The primary and secondary outcomes with repeated measurements across multiple time points were analyzed via linear mixed-effects models. Specifically, group, time, and the interaction of group-by-time were included as fixed effects, while the patient’s random intercepts were incorporated as random effects to account for differences in time. The group-by-time interaction term was tested first. If significant, between-group and within-group differences were tested at each time point, and analyses were adjusted for multiple comparisons using the Bonferroni test for differences in within-intervention from baseline (T0). Otherwise, the main effect of intervention was tested next. In the exploratory analysis, a post-hoc sensitivity analysis was performed first. Patients from both groups who had moderate-to-severe pain (NRS score ≥4) at T1 (the day of operation) were included, and the Mann–Whitney U test was used to compare pain-related outcomes between groups. Additionally, multiple linear regression analyses were applied for QoR-40 scores on postoperative day 1 (T2). Univariate linear regression identified factors with *p* < 0.1 that were included in the subsequent multivariate linear regression. Hypothesis tests were 2-sided with significance set at *p* < 0.05.

## Result

### Baseline characteristics

**Figure 1.**
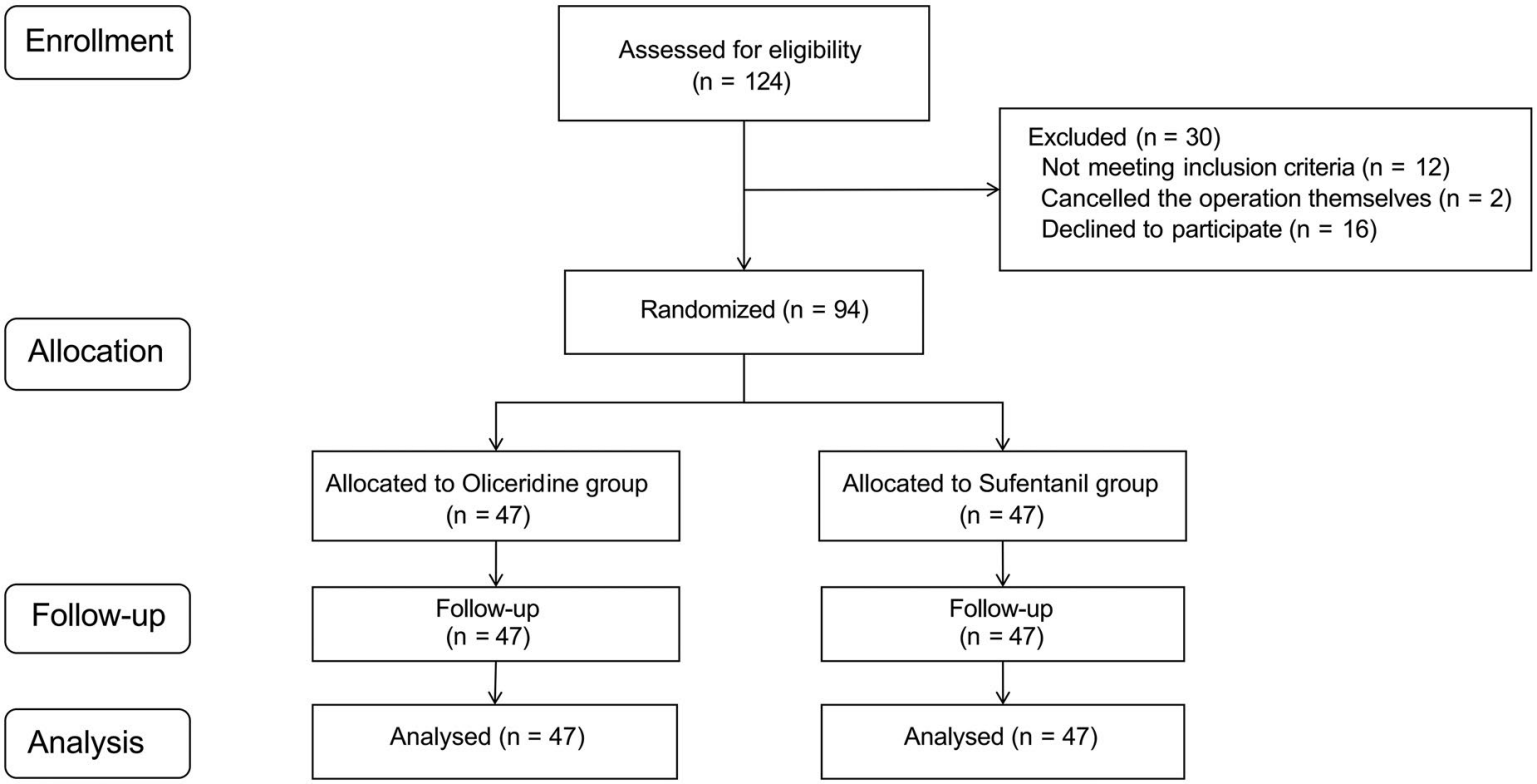
Clinical study flow chart.

In total, 124 participants were screened for eligibility. 16 patients refused to participate, 12 patients failed to meet inclusion criteria (8 patients because of abnormal electrocardiogram, 2 patients because of advanced gynecological tumours, 2 patients due to severe cardiovascular diseases), and 2 patients cancelled the operation. Finally, 94 patients were enrolled and randomly allocated to either the oliceridine group (*n* = 47) or the sufentanil group (*n* = 47), which completed trial recruitment (Figure 1). Table 1 shows the demographic and intraoperative characteristics, and there was no difference in baseline characteristics between the two groups.

**Table 1.** Baseline characteristics of study participants.

Characteristic	Oliceridine (*n* = 47)	Sufentanil (*n* = 47)	*P* value
Age, mean ± SD, year	49.5 ± 10.1	50.5 ± 10.6	0.855
Body mass index, mean ± SD, kg·m^-2^	23.6 ± 3.0	23.7 ± 3.3	0.286
Education level, No. (%)			0.727
Elementary school	15 (31.9)	15 (31.9)	
Middle school	16 (34.0)	20 (42.6)	
High school	4 (8.5)	4 (8.5)	
College graduate	12 (25.5)	8 (17.0)	
ASA, No. (%)			0.738
II	41 (87.2)	43 (91.5)	
III	6 (12.8)	4 (8.5)	
History of chemoradiotherapy, No. (%)	1 (2.1)	5 (10.6)	0.203
Comorbidities, No. (%)			0.897
Diabetes	2 (4.2)	4 (8.5)	
Hypertension	10 (21.3)	11 (23.4)	
Stroke	1 (2.1)	0 (0.0)	
Coronary artery disease	2 (4.3)	2 (4.3)	
ACCI, median (IQR)	2 (1, 2)	2 (1, 2)	0.571
Tumour classification, No. (%)			0.558
Endometrial cancer	28 (59.6)	23 (48.9)	
Cervical cancer	15 (31.9)	18 (38.3)	
Ovarian cancer	4 (8.5)	6 (12.8)	
Hemoglobin, mean ± SD, g·dL^-1^	12.0 ± 1.4	11.9 ± 1.8	0.113
QoR-40 score, mean ± SD	192.7 ± 3.0	192.3 ± 2.8	0.452
HADS-A score, median (IQR)	5 (3, 8)	6 (3, 8)	0.485
NRS score, median (IQR)	0 (0, 0)	0 (0, 0)	0.371
FRAIL, No. (%)			0.534
Robust	32 (68.1)	26 (55.3)	
Prefrail	13 (27.7)	18 (38.3)	
Frail	2 (4.3)	3 (6.4)	
Duration of operation, median (IQR), min	185 (170, 205)	185 (170, 205)	0.370
Duration of anesthesia, median (IQR), min	148 (116, 208)	165 (110, 232)	0.463
Infusion quantity, mean ± SD, mL	1936.8 ± 74.7	2146.8 ± 111.7	0.122
Remedial analgesia, No. (%)	8 (17.0)	11 (23.4)	0.441
Duration of hospitalization, median (IQR), d	13 (9, 16)	13 (10, 16)	0.884

ASA, American Society of Anesthesiologists Physical Status Classification; ACCI, Age-adjusted Charlson Comorbidity Index; QoR-40, the 40-item Quality of Recovery score; HADS-A, Hospital Anxiety and Depression Scale-Anxiety; NRS, Numeric rating scale; FRAIL, Fatigue, resistance, ambulation, illness and loss of weight.

### Primary outcomes

**Figure 2.**
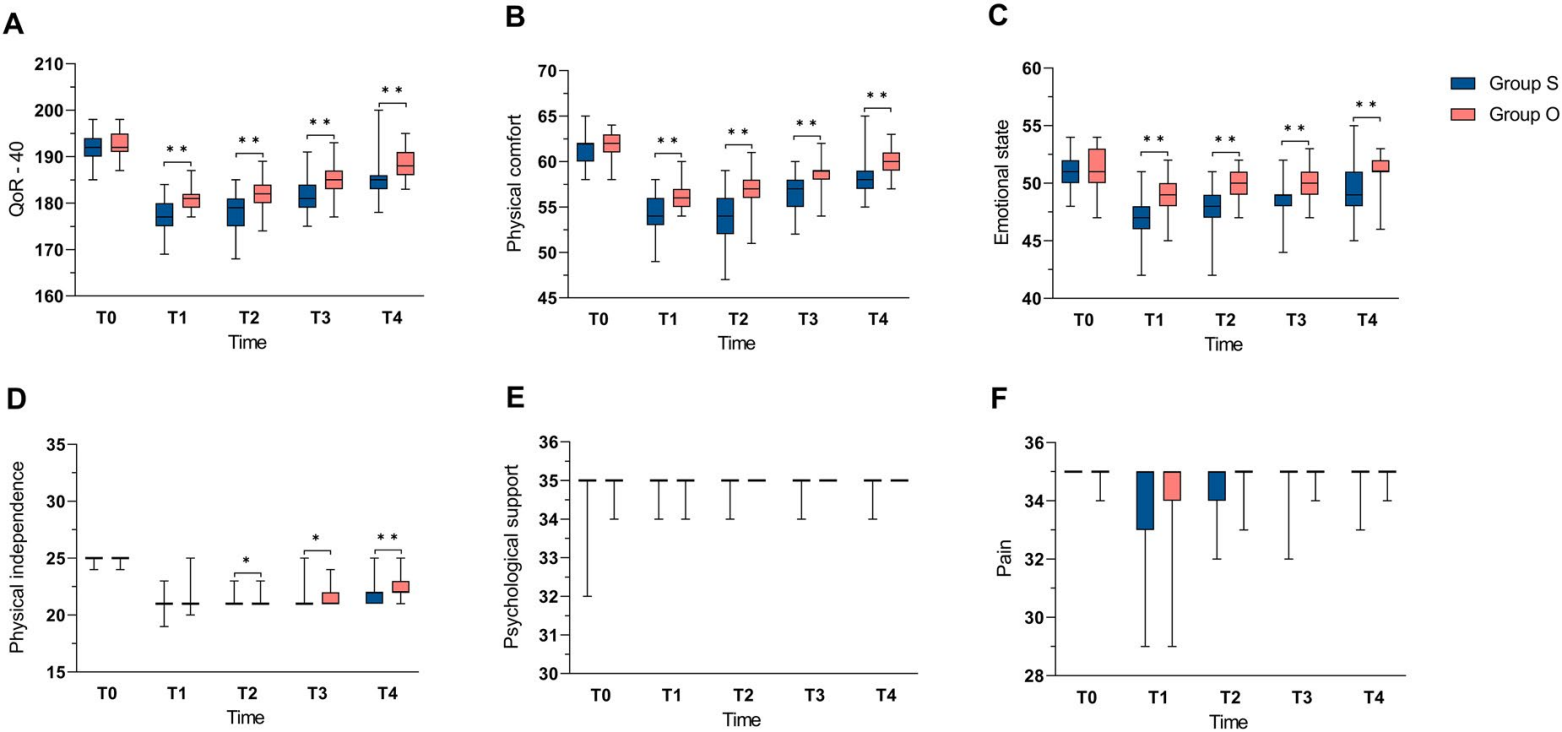
Comparison of QoR-40 scores and its 5 dimensions between groups. (A) Comparison of QoR-40 total scores, ranging from 40 to 200 points (higher scores indicate superior recovery quality); (B) Comparison of the Physical comfort subscale scores (12 to 60 points; 12 = severe persistent physical discomfort, 60 = absence of adverse physical symptoms); (C) Comparison of the Emotional state subscale scores (9 to 45 points; 9 = worst emotional status, 45 = optimal emotional condition); (D) Comparison of the Physical independence subscale scores (5 to 25 points; 5 = poorest physical independence, 25 = full physical independence); (E) Comparison of the Psychological support subscale scores (7 to 35 points; 7 = inadequate psychological support, 35 = optimal psychological support); (F) Comparison of the Pain subscale scores (7 to 35 points; 7 = persistent severe pain discomfort, 35 = complete absence of pain). Abbreviations: QoR-40, the 40-item Quality of Recovery score; T0, preoperative day; T1, operation day; T2, postoperative day 1; T3, postoperative day 2; T4, postoperative day 3; Group S, sufentanil group; Group O, oliceridine group. Data are presented as mean (standard deviation). *P < 0.05, **P < 0.01, versus Group S.

The mean QoR-40 score difference at postoperative day 1 (T2) was 5.4 points (95% CI: 4.0 to 6.8, *p* < 0.001; Cohen’s *d* = 1.53) with higher scores shown in patients in the oliceridine group (182.9 ± 3.1) compared with the sufentanil group (177.5 ± 3.9; Figure 2, Table 2).

**Table 2.** Comparison of Quality of Recovery-40 scores at multiple time points between groups.

	Oliceridine (*n* = 47)	Sufentanil (*n* = 47)	*P* value
**Quality of Recovery-40, mean ± SD**			
Drug-by-time interaction			<0.001
Main effect			<0.001
T0	192.7 ± 3.0	192.3 ± 2.8	0.504
T1	180.9 ± 2.4	177.0 ± 3.1	<0.001
T2	182.9 ± 3.1	177.5 ± 3.9	<0.001
T3	185.5 ± 3.0	181.3 ± 3.5	<0.001
T4	188.6 ± 2.9	184.9 ± 4.0	<0.001
**Physical comfort, mean ± SD**			
Drug-by-time interaction			<0.001
Main effect			<0.001
T0	61.8 ± 1.6	61.5 ± 1.5	0.427
T1	56.1 ± 1.3	54.2 ± 2.1	<0.001
T2	56.9 ± 2.0	54.2 ± 2.7	<0.001
T3	58.5 ± 1.6	56.5 ± 2.0	<0.001
T4	60.0 ± 1.4	58.3 ± 1.8	<0.001
**Emotional state, mean ± SD**			
Drug-by-time interaction			
Main effect			<0.001
T0	51.0 ± 1.8	50.9 ± 1.5	0.851
T1	49.0 ± 1.4	47.0 ± 1.8	<0.001
T2	49.6 ± 1.4	47.8 ± 1.9	<0.001
T3	50.3 ± 1.4	48.5 ± 1.5	<0.001
T4	51.3 ± 1.5	49.6 ± 1.9	<0.001
**Physical independence, mean ± SD**			
Drug-by-time interaction			0.553
Main effect			0.041
T0	25.0 ± 0.1	25.0 ± 0.2	0.559
T1	21.1 ± 0.7	21.0 ± 0.4	0.407
T2	21.2 ± 0.6	21.1 ± 0.3	0.046
T3	21.7 ± 0.9	21.4 ± 1.0	0.014
T4	22.4 ± 1.0	22.0 ± 1.2	0.007
**Psychological support, mean ± SD**			
Drug-by-time interaction			0.901
Main effect			0.198
T0	35.0 ± 0.2	34.9 ± 0.5	0.634
T1	35.0 ± 0.1	35.9 ± 0.2	0.559
T2	35.0 ± 0.0	35.0 ± 0.2	0.155
T3	35.0 ± 0.0	35.0 ± 0.2	0.155
T4	35.0 ± 0.0	35.0 ± 0.2	0.155
**Pain, mean ± SD**			
Drug-by-time interaction			0.156
Main effect			0.038
T0	34.9 ± 0.2	35.0 ± 0.0	0.080
T1	34.4 ± 1.3	34.0 ± 1.5	0.109
T2	34.8 ± 0.6	34.4 ± 1.0	0.093
T3	35.0 ± 0.2	34.8 ± 0.6	0.133
T4	35.0 ± 0.3	34.9 ± 0.2	0.235

T0, the day before the operation; T1, the day of operation; T2, postoperative day 1; T3, postoperative day 2; T4, postoperative day 3.

### Secondary outcomes

As shown in Table 2, the QoR-40 scores of patients in both groups within 3 days after operation were significantly lower than those before operation. The QoR-40 scores of both groups gradually increased from postoperative day 1. Specifically, the score in the oliceridine group reached 188.6 on postoperative day 3, while that in the sufentanil group was 184.9 (*p* < 0.001). Statistically significant differences were observed in the physical comfort and emotional state subscales of the QoR-40 between the two groups (*p* < 0.001). However, for physical independence, psychological support, and pain components, the linear mixed-effects model revealed no group-by-time interaction, and subsequent main effect analysis indicated no statistically significant differences between the two groups (*p* > 0.05).

The secondary outcomes analyzed using the linear mixed-effects model showed no interaction between group and time. Main effect analyses further indicated no statistically significant intergroup differences in NRS pain scores or HADS-A scores (*p* > 0.05; Table 3). However, FRAIL scores were significantly lower in the oliceridine group than in the sufentanil group on postoperative day 2 (T3) and postoperative day 3 (T4) (*p* < 0.05).

**Table 3.** Comparison of pain, anxiety and frailty between the two groups.

	Oliceridine (*n* = 47)	Sufentanil (*n* = 47)	*P* value
**NRS score, median (IQR)**			
Drug-by-time interaction			0.909
Main effect			0.439
T0	0.0 (0.0, 0.0)	0.0 (0.0, 0.0)	0.371
T1	1.0 (1.0, 2.0)	2.0 (1.0, 3.0)	0.120
T2	1.0 (1.0, 2.0)	1.0 (1.0, 2.0)	0.672
T3	1.0 (1.0, 1.0)	1.0 (1.0, 2.0)	0.209
T4	1.0 (1.0, 1.0)	1.0 (1.0, 1.0)	0.210
**HADS-A score, median (IQR)**			
Drug-by-time interaction			0.835
Main effect			0.147
T0	5.0 (3.0, 8.0)	6.0 (3.0, 8.0)	0.485
T1	5.0 (3.0, 8.0)	6.0 (4.0, 8.0)	0.246
T2	4.0 (4.0, 6.0)	5.0 (4.0, 8.0)	0.281
T3	4.0 (3.0, 7.0)	5.0 (4.0, 8.0)	0.215
T4	4.0 (3.0, 7.0)	5.0 (4.0, 8.0)	0.165
**FRAIL Index, median (IQR)**			
Drug-by-time interaction			0.555
Main effect			0.022
T0	0.0 (0.0, 1.0)	0.0 (0.0, 1.0)	0.249
T1	2.0 (2.0, 2.0)	2.0 (2.0, 2.0)	0.576
T2	2.0 (2.0, 2.0)	2.0 (2.0, 2.0)	0.241
T3	1.0 (1.0, 2.0)	2.0 (0.0, 1.0)	0.018
T4	1.0 (1.0, 1.0)	1.0 (1.0, 2.0)	0.037

NRS, Numeric Rating Scale; HADS-A, Hospital Anxiety and Depression Scale-Anxiety; FRAIL, Fatigue, Resistance, Ambulation, Illness and Loss of Weight; T0, the day before the operation; T1, the day of operation; T2, postoperative day 1; T3, postoperative day 2; T4, postoperative day 3.

### Post hoc outcomes

A further sensitivity analysis restricted to patients with moderate-to-severe pain on the day of the operation (T1) showed that the median pain score was 4 in both groups. The pain score gradually decreased, and no statistically significant difference was observed between the two groups within 3 days after the operation (*p* > 0.05; Table 4).

**Table 4.** Comparison of NRS scores of patients with moderate to severe postoperative pain at T1 between the two groups.

NRS score, median (IQR)	Oliceridine (*n* = 7)	Sufentanil (*n* = 8)	*P* value
T0	0.0 (0.0, 0.0)	0.0 (0.0, 0.0)	1.000
T1	4.0 (4.0, 4.0)	4.0 (4.0, 4.0)	1.000
T2	2.0 (1.0, 3.0)	2.5 (1.3, 3.8)	0.548
T3	1.0 (1.0, 2.0)	2.0 (2.0, 2.0)	0.178
T4	1.0 (1.0, 2.0)	1.5 (1.0, 2.0)	0.374

NRS, Numeric Rating Scale; T0, the day before the operation; T1, the day of operation; T2, postoperative day 1; T3, postoperative day 2; T4, postoperative day 3.

We performed univariable linear regression analyses to identify factors associated with the QoR-40 score on postoperative day 1 (T2). Variables with *p* < 0.10, including the treatment group, NRS pain score (T1) and postoperative nausea and vomiting (PONV) (T1), were entered into the subsequent multivariable linear regression model (Table 5). As shown in Table 6, treatment group and PONV were independently associated with the QoR-40 score at T2.

**Table 5.** Univariable linear regression analysis for postoperative recovery quality at T2.

Variable^a^	Coefficient (SE)	*t Ratio*	*P value*
Group	4.40 (0.76)	5.81	<0.001
Age	−0.01 (0.04)	−0.30	0.763
Education level	0.26 (0.40)	0.66	0.513
ASA classification	−1.30 (1.43)	−0.91	0.367
NRS score	−1.12 (0.57)	−1.97	0.052
PONV	−4.66 (0.76)	−6.15	<0.001
Hypoxia	−0.86 (1.59)	−0.54	0.590
Abdominal distension	−1.46 (1.20)	−1.22	0.227
Remedial analgesia	−0.82 (1.06)	−0.77	0.442
FRAIL	−0.53 (0.91)	−0.58	0.563

ASA, American Society of Anesthesiologists Physical Status Classification; NRS, Numeric Rating Scale; PONV, Postoperative Nausea and Vomiting; FRAIL, Fatigue, Resistance, Ambulation, Illness and Loss of Weight; T2, postoperative day 1.

^a^ Group (sufentanil group versus oliceridine group); Education level (Elementary school versus Middle school versus High school versus College graduate); ASA classification (II versus III); PONV (yes versus no); Hypoxia (yes versus no); Abdominal distension (yes versus no); Remedial analgesia (yes versus no); FRAIL (robust versus prefrail or frail).

**Table 6.** Multivariable linear regression analysis for postoperative recovery quality at T2.

Variable^a^	Coefficient (SE)	*t Ratio*	*P value*
Group	3.37 (0.88)	4.85	<0.001
NRS score	−0.54 (0.44)	−1.21	0.227
PONV	−3.54 (0.76)	−4.94	<0.001

*R* = 0.668; Adjusted *R*^2^ = 0.428; *F* = 24.159; *p* < 0.001.

NRS, Numeric Rating Scale; PONV, Postoperative Nausea and Vomiting; T2, postoperative day 1.

^a^ Group (sufentanil group versus oliceridine group); PONV (yes versus no).

### Adverse events

Regarding AEs, nausea was the most common in both groups: 25.5% (12/47) in the oliceridine group and 63.8% (30/47) in the sufentanil group (*p* < 0.05). Vomiting was the second most common AE, with no significant difference between the two groups (*p* = 0.111). Among respiratory AEs, hypoxaemia occurred in both groups (1 patient versus 7 patients; *p* = 0.019, Table 7). For serious adverse events (SAEs), 1 patient in the sufentanil group developed a postoperative wound infection, whereas 2 patients in the oliceridine group experienced SAEs: 1 patient with postoperative anal fistula and 1 patient with postoperative lymphatic leakage.

**Table 7.** Adverse events in the trial.

Events, No. (%)	Oliceridine (*n* = 47)	Sufentanil (*n* = 47)	*P* value
Postoperative nausea	12 (25.5)	30 (63.8)	<0.001
Postoperative vomiting	10 (21.3)	17 (36.2)	0.111
Dizziness	9 (19.1)	5 (10.6)	0.247
Fever	4 (8.5)	4 (8.5)	1.000
Hypoxaemia	1 (2.1)	7 (14.9)	0.019
Hypotension	1 (2.1)	1 (2.1)	1.000
Abdominal distension	5 (10.6)	8 (17.0)	0.370
Constipation	2 (4.3)	9 (19.1)	0.025
Wound infection	0 (0.0)	1 (2.1)	1.000
Postoperative anal fistula	1 (2.1)	0 (0.0)	1.000
Postoperative lymphatic leakage	1 (2.1)	0 (0.0)	1.000

## Discussion

This prospective randomized clinical trial demonstrated that, compared with sufentanil, oliceridine administered for PCIA may contribute to improving postoperative quality of recovery among patients undergoing laparoscopic gynecological tumour resection. Furthermore, oliceridine used for PCIA exhibited an analgesic efficacy comparable to that of sufentanil. Notably, the incidences of PONV and frailty were significantly lower in the oliceridine group; however, no significant difference was observed in postoperative anxiety between the two groups.

During the perioperative period, enhanced recovery after surgery (ERAS) guidelines require that clinicians focus not only on surgical disease outcomes but also on comprehensive postoperative recovery for surgical patients [36]. As a fundamental component of ERAS protocols, multimodal analgesia can alleviate patients’ surgical stress through appropriate pain management and rational pharmacotherapy, thereby facilitating early postoperative recovery [37]. In this trial, sufentanil served as the control; its notable advantages include broad clinical utility and excellent analgesic efficacy [38]. Existing studies have shown that oliceridine, a G-protein-biased μ-opioid receptor agonist, has analgesic efficacy primarily comparable to that of sufentanil [39]. However, no research has explored whether oliceridine may confer superior postoperative recovery quality due to fewer adverse effects. Recovery from surgery is a continuous process for patients, who require long-term management to achieve a relatively normal, independent state of optimal health [40]. From the perspective of patients, when compared to objective measures such as laboratory parameters and imaging studies, they typically prioritize their own perceptions of the disease and recovery progress [41]. Therefore, this study adopted the QoR-40 scale to evaluate patient’s postoperative recovery. As a validated tool for measuring recovery after general anaesthesia, the QoR-40 scale has well-established reliability and validity [42].

QoR-40 scores rose over time in both groups after the operation, and the QoR-40 scores of the oliceridine group were significantly higher than that of the sufentanil group. Although we observed a statistically significant between-group difference in QoR-40 scores of 5.4 at T2, this disparity failed to meet the predefined MCID of 6.3 points [35]. This modest intergroup gap may be attributed to two combined factors. First, all participants received standardized rehabilitation interventions; concurrent multiple recovery strategies diluted the incremental benefit attributable solely to the experimental intervention [43]. Second, a significant group-by-time interaction was observed for QoR-40 scores. The extent of score improvement over time varied between the two groups, which further narrowed the absolute total intergroup score gap. Between the two groups, differences were primarily observed in the comfort and emotional dimensions of the QoR-40, and no significant differences were noted in scores for physical independence or psychological support. For patients undergoing laparoscopic gynecological tumour resection, the recovery of independence, defined as the restoration of independent physical functions in daily life including working, communicating, writing and washing, generally requires a longer follow-up period [44,45]. This study concludes that early postoperative recovery quality in gynecological patients is predominantly driven by subjective experiences of physical comfort and surgical treatment results. Comparable scores were observed between groups in the pain domain of the QoR-40, indicating that oliceridine and sufentanil exert equivalent analgesic effects. However, the oliceridine group exhibited markedly better comfort levels, possibly owing to fewer respiratory and gastrointestinal ORAEs that alleviate physical discomfort [14].

Studies on the analgesic effect of oliceridine have shown its analgesic efficacy in rats to be 4- to 10-fold greater than that of morphine [39], with no evidence of opioid dependence observed to date [14]. In this trial, based on the morphine equivalent conversion, the equivalent ratio of sufentanil to oliceridine was determined to be 1:200 [46], and sufentanil at a dose of 3 μg/kg was chosen as the control. Based on preliminary experiment, this study ultimately selected oliceridine at 0.55 mg/kg for PCIA. This clinical study was conducted based on our institution’s standard 3-day postoperative analgesia protocol; accordingly, continuous monitoring of quality of recovery was performed over the first 3 postoperative days. The preliminary study revealed variable surgical end times among patients on the day of operation, which may cause observational bias. Therefore, postoperative day 1 was designated as the assessment time point for the primary outcome. A previous clinical trial comparing sufentanil with oliceridine in total knee arthroplasty patients found no significant between-group differences in postoperative NRS pain scores. Although our study differed substantially from that trial in terms of surgical population and analgesic regimens, we obtained consistent findings regarding pain scores [27]. On postoperative day 3 (T4), the median score in both groups was 1, indicating mild pain. Additionally, we performed a post hoc analysis of patients experiencing moderate-to-severe pain at T1 and observed that both groups exhibited mild pain at T2, a finding consistent with those of a previous prospective study on moderate to severe pain following abdominal surgery [47].

When analyzing FRAIL scores to evaluate between-group differences, no group-by-time interaction was observed between the two groups; the main effect analysis indicated that scores in the oliceridine group were superior to those in the sufentanil group, particularly on postoperative day 2 (T3) and day 3 (T4). Given the significant intergroup difference in the incidence of ORAEs, the more favorable tolerability of oliceridine likely largely accounts for the milder postoperative frailty seen in this group. No statistically significant difference was observed in perioperative anxiety scores between the two groups, which appears to contradict results from the emotional domain of the QoR-40. Perioperative anxiety has been defined as an emotional state [48], and the emotional domain of the QoR-40 encompasses items related to depression, sleep, anxiety, and other emotional factors. In this study, all patients exhibited baseline anxiety scores below eight points (non-anxiety) on the day before surgery, with further reductions in anxiety scores observed postoperatively. Nevertheless, we still hypothesize that, due to disease resolution and improved postoperative comfort, the emotional domain of the QoR-40 in the oliceridine group may be superior to that in the sufentanil group.

In this randomized controlled trial, AEs occurring within 3 days postoperatively were monitored. The incidence of nausea and vomiting was significantly higher in the sufentanil group relative to the oliceridine group, which aligns with the established mechanism of oliceridine. As a μ-opioid receptor biased agonist, it produces analgesic effects while inducing less β-arrestin recruitment, a property linked to fewer AEs [14,15]. Among the three SAEs recorded in this trial, none was judged to be attributable to oliceridine; all events were ascribed to disease progression and postoperative management issues.

This study has several limitations. First, this study only enrolled relatively healthy adult female patients from a single center, which limited result generalizability and may have created a ceiling effect. Future trials recruiting more heterogeneous populations may yield larger absolute between-group differences in outcome scores. Second, the optimal concentration of oliceridine for this study population was not specifically investigated but was derived from morphine equivalents and preliminary experiment. Therefore, its relative potency requires further investigation. Third, this study only observed patient recovery within 3 days postoperatively and did not assess the long-term effects of oliceridine on the study population. Fourth, the learning effect associated with repeated QoR-40 assessments was not analyzed or adjusted for.

## Conclusion

This prospective randomized controlled trial demonstrated that, compared with sufentanil, oliceridine improves the quality of early postoperative recovery in patients undergoing laparoscopic gynecological tumour resection. The two drugs demonstrated comparable analgesic efficacy, whereas oliceridine was associated with fewer adverse events and facilitated early rehabilitation. This study provides important evidence for the clinical application of novel postoperative analgesics.

## Supplementary Material

Clean_version_Figure_captions_revised.docx

CONSORT_Checklist.doc

## Data Availability

The data cannot be shared publicly because of participant privacy issues. Researchers can access the data by submitting a suitable proposal to the corresponding author, Fei Lin.

## References

[1] Li Y, Song W, Gao P, et al. Global, regional, and national burden of breast, cervical, uterine, and ovarian cancer and their risk factors among women from 1990 to 2021, and projections to 2050: findings from the global burden of disease study 2021. BMC Cancer. 2025;25(1):330. doi:10.1186/s12885-025-13741-9. 39988683 PMC11849330

[2] Fujiwara K, Connor SR, Fujiwara N, et al. The International Gynecologic Cancer Society consensus statement on palliative care. Int J Gynecol Cancer. 2024;34(8):1128–1132. doi:10.1136/ijgc-2024-005729. 38909991

[3] Keyvani V, Kheradmand N, Navaei ZN, et al. Epidemiological trends and risk factors of gynecological cancers: an update. Med Oncol. 2023;40(3):93. doi:10.1007/s12032-023-01957-3. 36757546

[4] Kenter GG, Greggi S, Vergote I, et al. Randomized phase III study comparing neoadjuvant chemotherapy followed by surgery versus chemoradiation in stage IB2-IIB cervical cancer: EORTC-55994. J Clin Oncol. 2023;41(32):5035–5043. doi:10.1200/jco.22.02852. 37656948

[5] Grassi L, Berardi MA, Ruffilli F, et al. Role of psychosocial variables on chemotherapy-induced nausea and vomiting and health-related quality of life among cancer patients: a European study. Psychother Psychosom. 2015;84(6):339–347. doi:10.1159/000431256. 26402426

[6] Gan TJ. Poorly controlled postoperative pain: prevalence, consequences, and prevention. J Pain Res. 2017;10:2287–2298. doi:10.2147/jpr.S144066. 29026331 PMC5626380

[7] Jiménez Cruz J, Kather A, Nicolaus K, et al. Acute postoperative pain in 23 procedures of gynaecological surgery analysed in a prospective open registry study on risk factors and consequences for the patient. Sci Rep. 2021;11(1):22148. doi:10.1038/s41598-021-01597-5. 34773057 PMC8590005

[8] Pirie K, Traer E, Finniss D, et al. Current approaches to acute postoperative pain management after major abdominal surgery: a narrative review and future directions. Br J Anaesth. 2022;129(3):378–393. doi:10.1016/j.bja.2022.05.029. 35803751

[9] Xu X, Tao Y, Yang Y, et al. Application of butorphanol versus sufentanil in multimode analgesia via patient controlled intravenous analgesia after hepatobiliary surgery: a retrospective cohort study. Drug Des Devel Ther. 2023;17:3757–3766. doi:10.2147/dddt.S433136. PMC10749102 38144418

[10] Stein C. Opioid receptors. Annu Rev Med. 2016;67(1):433–451. doi:10.1146/annurev-med-062613-093100. 26332001

[11] Fine P, Cheatle MD. Common adverse effects and complications of long-term opioid therapy. Pain Med. 2015;16(Suppl 1):S1–S2. doi:10.1111/pme.12925. 26350360

[12] Colvin LA, Bull F, Hales TG. Perioperative opioid analgesia—when is enough too much? A review of opioid-induced tolerance and hyperalgesia. Lancet. 2019;393(10180):1558–1568. doi:10.1016/s0140-6736(19)30430-1. 30983591

[13] Aubin HJ, Williams JM, Luquiens A. Opioid analgesics. Lancet. 2016;388(10045):656. doi:10.1016/s0140-6736(16)31268-5. 27533433

[14] Liang DY, Li WW, Nwaneshiudu C, et al. Pharmacological characters of oliceridine, a μ-opioid receptor G-protein-biased ligand in mice. Anesth Analg. 2019;129(5):1414–1421. doi:10.1213/ane.0000000000003662. 30044299 PMC12811984

[15] Goudra B. Oliceridine-opioid of the 21(st) century. Saudi J Anaesth. 2022;16(1):69–75. doi:10.4103/sja.sja_510_21. 35261592 PMC8846232

[16] Tan HS, Habib AS. Oliceridine: a novel drug for the management of moderate to severe acute pain—a review of current evidence. J Pain Res. 2021;14:969–979. doi:10.2147/jpr.S278279. 33889018 PMC8054572

[17] Schwienteck KL, Faunce KE, Rice KC, et al. Effectiveness comparisons of G-protein biased and unbiased mu opioid receptor ligands in warm water tail-withdrawal and drug discrimination in male and female rats. Neuropharmacology. 2019;150:200–209. doi:10.1016/j.neuropharm.2019.01.020. 30660628 PMC6476319

[18] Hammer GB, Khanna AK, Michalsky C, et al. Oliceridine exhibits improved tolerability compared to morphine at equianalgesic conditions: exploratory analysis from two phase 3 randomized placebo and active controlled trials. Pain Ther. 2021;10(2):1343–1353. doi:10.1007/s40122-021-00299-0. 34351590 PMC8586048

[19] Yi K, Sun W, Yu W, et al. Overview and prospects of the clinical application of oliceridine. Drug Des Devel Ther. 2025;19:5415–5430. doi:10.2147/dddt.S525471. PMC12209531 40599606

[20] Nafziger AN, Arscott KA, Cochrane K, et al. The influence of renal or hepatic impairment on the pharmacokinetics, safety, and tolerability of oliceridine. Clin Pharmacol Drug Dev. 2020;9(5):639–650. doi:10.1002/cpdd.750. 31697049 PMC7383509

[21] Beard TL, Michalsky C, Candiotti KA, et al. Oliceridine is associated with reduced risk of vomiting and need for rescue antiemetics compared to morphine: exploratory analysis from two phase 3 randomized placebo and active controlled trials. Pain Ther. 2021;10(1):401–413. doi:10.1007/s40122-020-00216-x. 33210266 PMC8119517

[22] Viscusi ER, Skobieranda F, Soergel DG, et al. APOLLO-1: a randomized placebo and active-controlled phase III study investigating oliceridine (TRV130), a G protein-biased ligand at the µ-opioid receptor, for management of moderate-to-severe acute pain following bunionectomy. J Pain Res. 2019;12:927–943. doi:10.2147/jpr.S171013. 30881102 PMC6417853

[23] Brzezinski M, Hammer GB, Candiotti KA, et al. Low incidence of opioid-induced respiratory depression observed with oliceridine regardless of age or body mass index: exploratory analysis from a phase 3 open-label trial in postsurgical pain. Pain Ther. 2021;10(1):457–473. doi:10.1007/s40122-020-00232-x. 33502739 PMC8119589

[24] Moss L, Hijma H, Demitrack M, et al. Neurocognitive effect of biased µ-opioid receptor agonist oliceridine, a utility function analysis and comparison with morphine. Anesthesiology. 2023;139(6):746–756. doi:10.1097/aln.0000000000004758. 37656771

[25] Tian Y, Hu J, Pan H, et al. Effect of oliceridine combined with sufentanil on patient-controlled intravenous analgesia in elderly patients after laparoscopic radical resection of rectal cancer: a prospective randomized controlled study. Drug Des Devel Ther. 2025;19:10033–10043. doi:10.2147/dddt.S553848. PMC12607661 41235138

[26] Hu N, Wang C, Zhang QQ, et al. Oliceridine versus sufentanil for postoperative recovery and opioid-related adverse events in patients undergoing thoracoscopic lobectomy: a randomized double-blind controlled trial. Drug Des Devel Ther. 2026;20:582482–582412. doi:10.2147/dddt.S582482. PMC13007703 41878674

[27] Liu X, Wang S, Chen X, et al. A comparison of oliceridine versus sufentanil for patient-controlled analgesia after total knee arthroplasty in elderly patients: a double-blinded randomized controlled study. Drug Des Devel Ther. 2026;20:587944–587910. doi:10.2147/dddt.S587944. PMC13050175 41939437

[28] Cepeda MS, Carr DB. Women experience more pain and require more morphine than men to achieve a similar degree of analgesia. Anesth Analg. 2003;97(5):1464–1468. doi:10.1213/01.Ane.0000080153.36643.83. 14570666

[29] Lefkowits C, Duska L. Opioid use in gynecologic oncology; balancing efficacy, accessibility and safety: an SGO clinical practice statement. Gynecol Oncol. 2017;144(2):232–234. doi:10.1016/j.ygyno.2016.11.015. 27988048

[30] Wheeler M, Oderda GM, Ashburn MA, et al. Adverse events associated with postoperative opioid analgesia: a systematic review. J Pain. 2002;3(3):159–180. doi:10.1054/jpai.2002.123652. 14622770

[31] Myles PS, Weitkamp B, Jones K, et al. Validity and reliability of a postoperative quality of recovery score: the QoR-40. Br J Anaesth. 2000;84(1):11–15. doi:10.1093/oxfordjournals.bja.a013366. 10740540

[32] Williamson A, Hoggart B. Pain: a review of three commonly used pain rating scales. J Clin Nurs. 2005;14(7):798–804. doi:10.1111/j.1365-2702.2005.01121.x. 16000093

[33] Zigmond AS, Snaith RP. The hospital anxiety and depression scale. Acta Psychiatr Scand. 1983;67(6):361–370. doi:10.1111/j.1600-0447.1983.tb09716.x. 6880820

[34] Abellan van Kan G, Rolland Y, Bergman H, et al. The I.A.N.A Task Force on frailty assessment of older people in clinical practice. J Nutr Health Aging. 2008;12(1):29–37. doi:10.1007/bf02982161. 18165842 PMC12892609

[35] Myles PS, Myles DB, Galagher W, et al. Minimal clinically important difference for three quality of recovery scales. Anesthesiology. 2016;125(1):39–45. doi:10.1097/aln.0000000000001158. 27159009

[36] Ljungqvist O, de Boer HD, Balfour A, et al. Opportunities and challenges for the next phase of enhanced recovery after surgery: a review. JAMA Surg. 2021;156(8):775–784. doi:10.1001/jamasurg.2021.0586. 33881466

[37] Carli F. Physiologic considerations of Enhanced Recovery After Surgery (ERAS) programs: implications of the stress response. Can J Anaesth. 2015;62(2):110–119. doi:10.1007/s12630-014-0264-0. 25501695

[38] Gepts E, Shafer SL, Camu F, et al. Linearity of pharmacokinetics and model estimation of sufentanil. Anesthesiology. 1995;83(6):1194–1204. doi:10.1097/00000542-199512000-00010. 8533912

[39] DeWire SM, Yamashita DS, Rominger DH, et al. A G protein-biased ligand at the μ-opioid receptor is potently analgesic with reduced gastrointestinal and respiratory dysfunction compared with morphine. J Pharmacol Exp Ther. 2013;344(3):708–717. doi:10.1124/jpet.112.201616. 23300227

[40] Lee L, Tran T, Mayo NE, et al. What does it really mean to “recover” from an operation? Surgery. 2014;155(2):211–216. doi:10.1016/j.surg.2013.10.002. 24331759

[41] Bowyer A, Royse C. Approaches to the measurement of post-operative recovery. Best Pract Res Clin Anaesthesiol. 2018;32(3–4):269–276. doi:10.1016/j.bpa.2018.02.001. 30522717

[42] Gornall BF, Myles PS, Smith CL, et al. Measurement of quality of recovery using the QoR-40: a quantitative systematic review. Br J Anaesth. 2013;111(2):161–169. doi:10.1093/bja/aet014. 23471753

[43] Soffin EM, Beckman JD, Tseng A, et al. Enhanced recovery after lumbar spine fusion: a randomized controlled trial to assess the quality of patient recovery. Anesthesiology. 2020;133(2):350–363. doi:10.1097/aln.0000000000003346. 32433277

[44] Lee WK, Kim MS, Kang SW, et al. Type of anaesthesia and patient quality of recovery: a randomized trial comparing propofol-remifentanil total i.v. anaesthesia with desflurane anaesthesia. Br J Anaesth. 2015;114(4):663–668. doi:10.1093/bja/aeu405. 25500679

[45] Niu Z, Gao X, Shi Z, et al. Effect of total intravenous anesthesia or inhalation anesthesia on postoperative quality of recovery in patients undergoing total laparoscopic hysterectomy: a randomized controlled trial. J Clin Anesth. 2021;73:110374. doi:10.1016/j.jclinane.2021.110374. 34090183

[46] Walker G. The opioid crisis: a 21st century pain. Drugs Today (Barc). 2018;54(4):283–286. doi:10.1358/dot.2018.54.4.2812620. 29869649

[47] Singla N, Minkowitz HS, Soergel DG, et al. A randomized, phase IIb study investigating oliceridine (TRV130), a novel µ-receptor G-protein pathway selective (μ-GPS) modulator, for the management of moderate to severe acute pain following abdominoplasty. J Pain Res. 2017;10:2413–2424. doi:10.2147/jpr.S137952. 29062240 PMC5638571

[48] Ford JL, Ildefonso K, Jones ML, et al. Sport-related anxiety: current insights. Open Access J Sports Med. 2017;8:205–212. doi:10.2147/oajsm.S125845. 29138604 PMC5667788

