# Supplementary material for: Oliceridine used for patient-controlled analgesia on postoperative quality of recovery in patients undergoing laparoscopic gynecological tumour resection: a randomized clinical trial
Source: Ann Med. 2026 Aug 12;58(1):2714613. doi: 10.1080/07853890.2026.2714613 (PMC13470910; doi:10.1080/07853890.2026.2714613)
Supplement: Supplementary file 1 [file IANN_A_2714613_SM0275.docx]

Figure 1. Clinical study flow chart.

Figure 2. Comparison of QoR-40 scores and its 5 dimensions between groups. (A) Comparison of QoR-40 total scores, ranging from 40 to 200 points (higher scores indicate superior recovery quality); (B) Comparison of the Physical comfort subscale scores (12 to 60 points; 12 = severe persistent physical discomfort, 60 = absence of adverse physical symptoms); (C) Comparison of the Emotional state subscale scores (9 to 45 points; 9 = worst emotional status, 45 = optimal emotional condition); (D) Comparison of the Physical independence subscale scores (5 to 25 points; 5 = poorest physical independence, 25 = full physical independence); (E) Comparison of the Psychological support subscale scores (7 to 35 points; 7 = inadequate psychological support, 35 = optimal psychological support); (F) Comparison of the Pain subscale scores (7 to 35 points; 7 = persistent severe pain discomfort, 35 = complete absence of pain).

Abbreviations: QoR-40, 40-item Quality of Recovery questionnaire; T0, preoperative day; T1, operation day; T2, postoperative day 1; T3, postoperative day 2; T4, postoperative day 3; Group S, sufentanil group; Group O, oliceridine group. Data are presented as mean (standard deviation). **P* < 0.05, ***P* < 0.01, versus Group S.
